# The Mediating Role of Self-Criticism in the Relationship Between Coaches’ Leadership Styles and Disordered Eating in Athletes

**DOI:** 10.3390/nu17030427

**Published:** 2025-01-24

**Authors:** Carol Coelho, Diane Oliveira, Catarina Branco, António Rui Gomes, Eva Conceição, Paulo P. P. Machado, Sónia Gonçalves

**Affiliations:** 1 Psychology Research Center (CIPsi), School of Psychology, University of Minho, 4710-057 Braga, Portugal; pg51478@alunos.uminho.pt (D.O.); pmachado@psi.uminho.pt (P.P.P.M.); sgoncalves@psi.uminho.pt (S.G.); 2 Center for Psychology, Faculty of Psychology and Education Sciences, University of Porto, 4200-135 Porto, Portugal; econceicao@fpce.up.pt

**Keywords:** coach leadership styles, coach–athlete relationship, self-criticism, disordered eating, sports psychology

## Abstract

Background/Objectives: Athletes are particularly vulnerable to developing eating disorders, which can negatively impact both health and athletic performance. The coach–athlete relationship plays a central role in athletes’ development and well-being. However, little is known about how coaches’ leadership styles relate to athletes’ self-criticism and disordered eating. Therefore, this study aimed to assess the relationship between athletes’ perceptions of their coach’s leadership style and their own self-criticism and disordered eating. Methods: A total of 150 athletes from team ball sports, aged 18 to 43 years (M = 25.0, SD = 6.0), completed self-report measures. Results: 27.3% of the participants were at heightened risk of developing eating disorders, and 38.7% lacked access to nutritional or psychological support within their clubs. Significant positive associations were found between coaches’ negative feedback style and athletes’ self-criticism and disordered eating, and between passive management style and athletes’ self-criticism and disordered eating. Moreover, athletes’ self-criticism fully mediated the relationship between these leadership styles and athletes’ disordered eating. Additionally, coaches’ vision, inspiration, and individualization styles were related to decreased self-criticism in athletes. Conclusions: Coaches’ leadership styles are significantly related to athletes’ psychological and nutritional health. This research has important implications for promoting healthier practices in sports settings.

## 1. Introduction

Athletes experience general societal pressures concerning appearance from peers, family, and the media, alongside sport-specific demands that intensify their attention to and commitment to their bodies, as their performance is closely tied to their physical condition [1]. Compared to non-athletes, athletes are at greater risk of developing disordered eating and eating disorders [2]. Systematic reviews [2,3,4] have identified young female and lean sports athletes as a higher-risk population for eating disorders among athletes. This can be attributed to the higher societal standards placed on women’s bodies, and by the strong association between low weight and performance in lean sports [3]. However, disordered eating and eating disorders are prevalent across athletes’ genders, ages, and sports types [2], affecting also male athletes [5], older athletes [3], and athletes from non-lean sports, such as team ball sports [6,7].

Eating disorders are characterized by significant disturbances in eating and eating-related behavior that negatively affect physical health and/or psychosocial functioning [8]. Disordered eating, a less severe manifestation along the eating disorder continuum, encompasses attitudes and behaviors such as preoccupation with weight and shape, and engagement in inappropriate weight control behaviors [9]. Disordered eating can impair both health and sport performance [10,11], leading to the development of eating disorders [12]. Several factors have been identified as contributing to athletes’ vulnerability to disordered eating, including messages from coaches and teammates, mandatory weigh-ins, sports body stereotypes, revealing uniforms, the belief that weight affects performance, high nutritional and water requirements, and the considerable psychological, physical, and time investment in training and competition [1,2,5,13,14,15].

The coach–athlete relationship is central to athletes’ development and progress [15] and influences athletes’ motivation and engagement [16]. Despite the growing interest in disordered eating among athletes [14], research on the role of coaching in these behaviors remains limited, making it an important area for further investigation [7,17]. Certain factors, such as coach–athlete attachment style and coaches’ pressures or messages about weight, shape, and eating, have a more direct and observable connection to athletes’ disordered eating [18,19,20,21,22]. In contrast, less overt factors, like coaches’ leadership styles, may also significantly contribute to athletes’ vulnerability to these behaviors, warranting further investigation.

To date, and to the best of our knowledge, only one empirical study has directly examined the association between coaches’ leadership styles and athletes’ disordered eating [23]. This study revealed that disordered eating amongst adolescent female volleyball players was positively associated with an autocratic coaching style and lack of coaches’ social support. Conversely, no significant associations were found between athletes’ disordered eating and the other leadership styles that were studied, which included training instruction, reinforcement, and democratic leadership [23].

Further exploration is needed to deepen our understanding of the interplay between coaches’ leadership styles and athletes’ disordered eating, particularly among male and adult populations and across different sports. Additionally, the potential influence of other leadership styles—such as vision, inspiration, individualization, positive feedback, negative feedback, active management, and passive management—remains unexplored. Moreover, delving into psychological variables related to eating disorders among athletes could provide deeper insights into the mechanisms linking coaches’ leadership styles to athletes’ disordered eating [17].

One such variable is self-criticism, a maladaptive coping mechanism characterized by negative self-judgment and self-scrutiny in response to perceived flaws and inadequacies [24]. Self-criticism has been associated with higher levels of disordered eating behaviors [25,26]. Specifically, among athletes, self-criticism is linked to greater disordered eating [27] and perceptions of more critical attitudes from coaches [28]. To the best of our knowledge, no study has investigated the mediating role of athletes’ self-criticism in the relationship between coaches’ leadership styles and athletes’ disordered eating. Addressing the aforementioned gaps is essential to comprehensively understanding how coaches’ leadership styles are related to athletes’ vulnerability to disordered eating, which can ultimately inform prevention programs [29].

**Figure 1:**
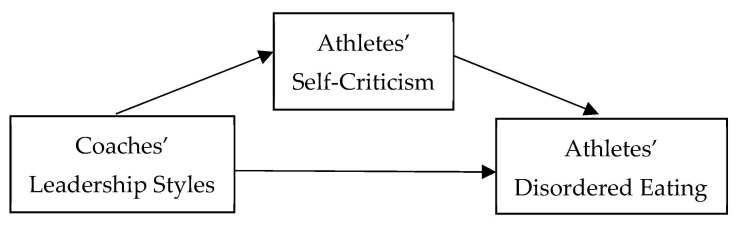
Mediating role of athletes’ self-criticism in the relationship between coaches’ leadership styles and athletes’ disordered eating. Note: In this hypothesized model, coaches’ leadership styles impact athletes’ self-criticism, which in turn influences disordered eating behaviors. Understanding this relationship can guide targeted interventions and prevention programs to reduce disordered eating in athletes.

Therefore, the present study aimed to evaluate the relationship between athletes’ perceptions of their coach’s leadership style and their self-criticism and disordered eating. In addition, it sought to examine the mediating role of self-criticism in the link between coaches’ leadership styles and athletes’ disordered eating. We hypothesized that coaches’ negative feedback and passive management styles would be positively associated with athletes’ disordered eating, and that coaches’ vision, inspiration, instruction, individualization, support, positive feedback, and active management styles would be negatively related to athletes’ disordered eating. Further, we expected self-criticism to serve as a significant mediator in these relationships (see Figure 1).

## 2. Materials and Methods

### 2.1. Participants

The sample consisted of 150 athletes currently competing at the senior level in Portugal, with 97 (64.7%) identifying as female and 53 (35.3%) as male. Participants’ ages ranged from 18 to 43 years (*M* = 25, *SD* = 6.0), with an average body mass index (BMI) of 23.1 (*SD* = 2.1). A total of 126 athletes (84%) exhibited BMI values classified as within the normal weight range, 23 athletes (15.3%) were categorized as overweight, and one athlete (0.7%) presented with Grade I obesity. The sample included athletes of various nationalities, mostly Portuguese (*n* = 134, 89.3%). Athletic experience ranged from 1 to 35 years (*M* = 13.9, *SD* = 6.6). On average, athletes trained for 2 h and 30 min (*SD* = 1.4) per day, with the majority (85.3%) training four or more days per week. Regarding sports representation, the distribution was as follows: volleyball (*n* = 67, 44.7%), futsal (*n* = 41, 27.3%), football (*n* = 27, 18%), handball (*n* = 10, 6.7%), and basketball (*n* = 5, 3.3%). All athletes were registered competitors in major leagues. Of these, 39 athletes (26%) were classified as professional, while the remaining 111 (74%) were amateur.

### 2.2. Measures

Sociodemographic and Sport-Related Questionnaire: this collected information regarding age, gender, weight, height, nationality, practiced sport, years of experience in the sport, average training hours per day, number of training days per week, and availability of nutritional and psychological services at the club.

Eating Disorder Examination Questionnaire (EDE-Q [30], Portuguese version [31]): this assessed disordered eating behaviors, with higher scores indicating greater disordered eating severity. The global score demonstrated good internal consistency, with a Cronbach’s alpha of α = 0.87.

Self-Criticism Subscales of the Forms of Self-Criticizing and Reassuring Scale (FSCRS [24], Portuguese version [32]): this evaluated self-criticism through two dimensions, the Inadequate Self and the Hated Self subscales. Higher scores on the Inadequate Self subscale reflect greater self-criticism focused on feelings of inadequacy or failure (e.g., “I am easily disappointed with myself”, “I feel I deserve to be criticized”), while higher scores on the Hated Self subscale indicate greater self-criticism characterized by self-hatred or self-directed anger (e.g., “I do not like being me”, “I have become so angry with myself that I want to hurt or punish myself”). The subscales demonstrated excellent (α = 0.90) and acceptable (α = 0.76) internal consistency, respectively.

Multidimensional Scale of Leadership in Sport (MSLS [33]): this assessed athletes’ perceptions of their coaches’ leadership style through nine subscales: Vision (e.g., “My coach defines a positive vision for my future”), Inspiration (e.g., “My coach encourages me to work the best I can”), Instruction (e.g., “My coach tells me what to do and how to do it, facilitating my progress in this sport”), Individualization (e.g., “My coach respects me as a person and not as just one more team member”), Support (e.g., “My coach helps me when I have a personal problem”), Positive Feedback (e.g., “My coach demonstrates satisfaction when I perform well”), Negative Feedback (e.g., “My coach gets irritated with me when I don’t do things as planned”), Active Management (e.g., “My coach encourages me to give suggestions about what to do in training/competitions”), and Passive Management (e.g., “My coach allows problems to go on before doing something about it”). The scale consists of 36 items, answered on a Likert scale with five response options (1 = Never, 5 = Always). Higher scores in each subscale indicate a greater perceived frequency of the respective coach behaviors. Gomes and Resende [33] found acceptable internal consistency for items (all α > 0.70), with confirmatory factor analysis indicating acceptable fit indices for the nine-factor structure, which can be divided into three domains of leadership: transformational (Vision, Inspiration, Instruction, Individualization and Support), transactional (Positive and Negative Feedback), and decision-making (Active and Passive Management). In this study, the subscales demonstrated acceptable to excellent internal consistency: Vision (α = 0.93), Inspiration (α = 0.86), Instruction (α = 0.86), Individualization (α = 0.86), Support (α = 0.81), Positive Feedback (α = 0.84), Negative Feedback (α = 0.83), Active Management (α = 0.85), and Passive Management (α = 0.72).

### 2.3. Procedure

This study was conducted in accordance with the Declaration of Helsinki and received approval from the Ethics Committee for Research in Social and Human Sciences of the University of Minho (CEICSH 152/2023). Following approval, a combination of convenience and snowball sampling methods was used. Clubs were contacted to explain the study’s objectives and request data collection permission. Before participating, the athletes were informed about confidentiality and their right to withdraw without any repercussions through an informed consent process. Data were collected using Google Forms at the clubs’ facilities, without the presence of coaches. In cases where clubs did not respond, athletes were contacted via email or social media and invited to complete the questionnaire at their convenience. Data collection took place three to four months after the start of the sports season, allowing time for athletes and coaches to become familiar with one another. The questionnaires included both Portuguese and English versions to accommodate foreign athletes competing in the teams.

### 2.4. Data Analysis

Data treatment was conducted using Microsoft Excel. Subsequently, the data were exported to IBM SPSS Statistics (Version 29), where frequency, internal consistency, correlation and mediation analyses were performed. The significance level was set at *p* < 0.05. Skewness (sk) and kurtosis (Ku) values were within the recommended ranges (|sk| < 3 and |Ku| < 10; [34]), confirming the normality of the data. Pearson correlation coefficients were used to evaluate the correlations among the interest variables (disordered eating, self-criticism and coaches’ leadership style). Next, mediation analyses were conducted between variables that showed significant correlations, with disordered eating as the dependent variable, self-criticism subscales as mediators, and coaches’ leadership styles as independent variables.

## 3. Results

### 3.1. Disordered Eating Prevalence and Availability of Support Services

Regarding disordered eating behaviors, 41 athletes (27.3%) scored above the EDE-Q cut-off based on Portuguese norms [31], indicating significant impairment or distress related to body and eating concerns. In terms of the availability of psychological and nutritional services, 72 athletes (48%) reported that their clubs provided both services, while 10 athletes (6.7%) indicated the presence of only psychological services, another 10 athletes (6.7%) reported the existence of only nutritional services, and 58 athletes (38.7%) stated that neither service was available at their respective clubs.

### 3.2. Association Between Studied Variables

The results of the correlation analyses are presented in Table 1. Overall, athletes’ disordered eating was positively associated with both self-criticism subscales and with coaches’ negative feedback and passive management styles. Moreover, the Inadequate Self subscale of self-criticism was positively associated with coaches’ negative feedback style, while the Hated Self subscale of self-criticism was positively associated with their passive management style. Additionally, The Hated Self subscale was negatively related to coaches’ vision, inspiration, and individualization styles.

### 3.3. Mediation Analyses

Given the significant positive correlations between the coaches’ negative feedback style, athletes’ disordered eating, and athletes’ self-criticism (Inadequate Self subscale), a mediation analysis was conducted to examine whether this form of self-criticism mediates the relationship between negative feedback and disordered eating. The results, presented in Table 2, indicate that negative feedback predicts disordered eating (total effect), and that self-criticism (Inadequate Self subscale) mediates this association (indirect effect), accounting for 49.5% of negative feedback’s effect on disordered eating. Moreover, the lack of a significant direct effect of negative feedback on disordered eating implies that self-criticism fully mediates this relationship. These findings suggest that negative feedback from coaches may increase athletes’ self-criticism, contributing to greater disordered eating behaviors.

Considering the significant positive correlations between coaches’ passive management style, athletes’ disordered eating, and athletes’ self-criticism (Hated Self subscale), a mediation analysis was performed to determine whether this form of self-criticism mediates the association between passive management and disordered eating. The findings, shown in Table 3, demonstrate that passive management predicts disordered eating (total effect), and that self-criticism (Hated Self subscale) mediates this relationship (indirect effect), explaining 54.4% of passive management’s effect on disordered eating. Furthermore, the absence of a significant direct effect suggests complete mediation by self-criticism. These results indicate that passive management may heighten athletes’ self-criticism, increasing their disordered eating behaviors.

## 4. Discussion

This study aimed to assess the association between athletes’ perceptions of their coach’s leadership style and their self-criticism and disordered eating. It also sought to analyze the potential mediating role of self-criticism in the relationship between coaches’ leadership styles and athletes’ disordered eating.

Regarding the prevalence of disordered eating behaviors, 27.3% of the participants scored above the EDE-Q global score cut-off established for the Portuguese population [31]. According to Lichtenstein et al. [14], the EDE-Q has demonstrated strong external validity, with high sensitivity and specificity in assessing eating disorder symptoms’ prevalence. This evidence supports the reliability of our findings, which indicate that a considerable proportion of athletes from team ball sports may experience significant impairment or distress related to their body and eating concerns, placing them at heightened risk of developing eating disorders. Interestingly, our findings revealed a higher prevalence of disordered eating behaviors among athletes compared to a meta-analysis that combined data from several countries (19.2%, [35]), including Portugal (17.7%, [36]). This discrepancy could reflect an overestimation due to our smaller sample, differences in sampling techniques, or the focus on a specific sports category—team ball sports. Team sports often involve more group dynamics, which might amplify body comparison, which has been linked to an increased drive for thinness and dietary restraint [37]. Alternatively, it may indicate a genuine increase in disordered eating behaviors among athletes from Portugal.

Notably, nearly 40% of participants reported no access to nutritional or psychological services at their respective clubs, and approximately 15% had access to only one of these services. The lack of comprehensive support systems may leave athletes vulnerable to developing disordered eating behaviors, as these services are critical for both prevention and treatment. These results highlight the pressing need to improve access to nutritional and psychological support within sports clubs to address disordered eating behaviors effectively [38].

Although some athletes perceive self-criticism as a means for improvement and goal attainment [39], it has been found to induce negative emotional responses after setbacks in goal pursuit [40] and hinder psychological flourishing in sports [41]. The correlation analyses revealed that self-criticism is related to greater disordered eating among athletes, consistent with previous research [27], suggesting that while self-criticism may seem like a productive motivator, it might be linked to maladaptive eating behaviors. This finding underscores the need for targeted interventions or educational programs that help athletes manage self-criticism in more constructive ways, such as shifting from self-criticism to self-compassion [42].

Moreover, the correlation analyses showed that athletes who perceive their coaches’ leadership style as involving negative feedback and passive management are more likely to exhibit higher levels of self-criticism and disordered eating. Thus, coaches who convey negative feedback and employ a passive management style may contribute to athletes adopting self-criticism and developing disordered eating behaviors. Regarding negative feedback, both quantitative and qualitative research have found that excessive criticism from coaches can foster self-criticism in athletes [28,42]. Furthermore, Barić and Erdeljac [18] showed that athletes perceived greater pressure about food and weight when their coaches exhibited negative behaviors, including negative feedback. Regarding passive management, the lack of feedback, engagement, guidance, and active problem-solving may deprive athletes of the necessary support to navigate challenges effectively. Without this structure, athletes may experience uncertainty [33]. Intolerance of uncertainty, which has been identified as a transdiagnostic vulnerability and maintenance factor for disordered eating [43], is likely to play a significant role in this context. Thus, athletes may experience distress when faced with uncertainty, leading to increased self-criticism. As a result, athletes may engage in maladaptive coping strategies, such as disordered eating, as a means to deal with their discomfort.

On the other hand, perceptions of coaches’ vision, inspiration, and individualization styles were associated with lower levels of self-criticism. By presenting an optimistic vision of athletes’ future, demonstrating positive expectations and behaviors toward promoting athletes’ success, and considering athletes’ individual needs—key components of what is known as transformational leadership—coaches may model these positive behaviors and also create a more positive and inclusive sports environment [33]. In fact, Frentz et al. [42] found that when coaches prioritized athletes’ well-being and needs and fostered open communication, athletes were more likely to adopt a self-compassionate stance rather than a self-critical one.

Interestingly and contrary to our hypothesis, coaches’ vision, inspiration, and individualization styles were not significantly correlated to athletes’ disordered eating. It is possible that the cross-sectional nature of the study, the use of self-report measures, and the relatively small sample size may have influenced these findings. Additionally, more negative leadership styles—such as negative feedback and passive management—may be more closely related to athletes’ disordered eating and self-criticism than more positive leadership styles. Another unexpected result that may be explained by these factors was the lack of significant associations between athletes’ self-criticism and disordered eating and their perceptions of coaches’ instruction, support, active management, and positive feedback. Specifically, the lack of association between coaches’ instruction and athletes’ disordered eating, may be due to the fact that teaching and improving technical skills does not significantly impact disordered eating behaviors (as found by Fortes et al. [23]), since these skills do not directly involve eating and body image concerns. Additionally, the presence of opposing items (such as passive management and negative feedback) may have reduced the statistical power of the results for positive feedback (as found by Fortes et al. [23]) and active management.

The mediation analyses revealed that the relationship between athletes’ disordered eating and their coaches’ negative feedback and passive management styles is fully mediated by self-criticism. Therefore, when coaches constantly provide negative feedback, athletes may internalize this criticism, leading to feelings of inadequacy. In addition, a passive management style, characterized by a lack of guidance and support, may increase feelings of uncertainty. Consequently, athletes may experience increased self-criticism, which has been linked to unhealthy perfectionism and psychological distress, namely anxiety, stress and depression [44]. In an attempt to deal with these emotions, and given the belief most athletes have that weight affects performance [1], athletes may engage in disordered eating behaviors. Although no study has specifically analyzed the mediating role of self-criticism in this context, Shanmugan et al. [20] found that coach–athlete relationships marked by decreased support and increased conflict were associated with higher levels of disordered eating in athletes, through the mediation of self-critical perfectionism. Our results, along with those of Shanmugan et al. [20], suggest that self-criticism and related tendencies are key mechanisms in the relationship between coach-athlete dynamic and athletes’ disordered eating. From a practical perspective, coaches should be mindful of how their feedback style affects athletes’ self-perception, providing constructive feedback. They must also actively engage with their athletes by providing clear directions, fostering open communication, and offering consistent support. Further, our correlation and mediation findings emphasize the need for targeted interventions and training/educational programs that help coaches develop more positive leadership styles, which in turn can help mitigate athletes’ self-criticism and disordered eating.

### 4.1. Limitations and Recommendations for Future Research

This study has some limitations that should be addressed, along with recommendations for future research. First, the small number of participants may have reduced statistical power in our analysis, prompting some results to be statistically insignificant. The athletes’ age range in this study may also be a limitation, since experiences related to disordered eating, self-criticism, and coaching methods may be quite different. Future studies should include larger samples, allowing comparisons according to variables such as age, gender, BMI, type of sport, years of experience, and level of proficiency. This would help address a limitation of this study, namely, the lack of control over potential variances in how athletes of different ages, genders, BMIs, sports types, years of experience, and levels of proficiency experience these behaviors and respond to coaching methods. In addition, potential underlying variables should be included, some of which have been linked to coaches’ leadership style—such as perfectionism [45] and emotion regulation [46]—and others that remain unexplored in this context, such as self-compassion, negative affect, and intolerance of uncertainty. These variables may act as moderators or mediators in the relationship between coaches’ leadership styles and athletes’ self-criticism and disordered eating.

Another limitation in our work is the use of cross-sectional methodology, which prevents conclusions regarding causality. Therefore, our results should be considered cautiously, reflecting associations rather than causal relationships. Furthermore, our study considers athletes’ perspectives using self-report measures. The reliance on self-reporting is also a limitation due to the possibility of social desirability influencing the results. For example, although we ensured anonymity, athletes may have feared the consequences of evaluating their coaches’ attitudes and disclosing information about their mental health. Thus, future research can benefit from longitudinal, qualitative, and observational studies, including both athletes’ and coaches’ perspectives. More specifically, longitudinal studies allow examining causality and temporal dynamics between leadership styles, self-criticism, and disordered eating. Furthermore, according to Voelker et al. [22], studying coaches’ perspectives will position coaches as targeted agents of change to effectively address disordered eating in athletes.

Although we measured disordered eating with an instrument that has demonstrated high external validity in identifying eating disorder diagnoses [14], future studies could be improved by employing instruments specifically validated for the athletic population, as stated by Flatt et al. [47]. Lastly, future research should examine whether athletes with access to support services utilize them within the club, seek external support, or do not engage with these services. It would also be worthwhile to explore whether athletes without access to these services at their clubs seek private support and to assess the impact of both in-club and external nutritional and psychological services on their mental and physical health.

### 4.2. Implications

Findings from this study can contribute to a broader understanding and conceptualization of disordered eating in athletes by highlighting the influence of coaches’ leadership styles and athletes’ self-criticism within this context. This research also adds to the training of coaches by emphasizing the importance of providing constructive feedback, engaging proactively with athletes, and employing effective leadership techniques that foster a positive and inclusive environment, particularly transformational leadership styles [33].

Furthermore, this study advocates for club leaders to implement and strengthen psychological and nutritional services, which can enhance athletes’ physical and mental well-being. Specifically, athletes should be screened for disordered eating and receive immediate access to support tailored to their needs [47]. As noted by Kokko [48], investing in health promotion allows sports clubs to not only enhance their athletes’ well-being, but also support the clubs’ success and meet societal expectations.

Finally, this research underscores the need for both intervention and educational programs. Specifically, educational initiatives should focus on increasing awareness of the risk factors and consequences of disordered eating, as well as reducing stigma surrounding sport psychology, mental health, and disordered eating for athletes, coaches, staff members, and the athletes’ support network [10,12,49,50]. Additionally, prevention and intervention programs should aim to improve coaches’ leadership styles and behaviors, assist coaches and athletes in building stronger relationships, and promote more open communication [15,41,51]. Furthermore, these programs should encourage self-compassion and foster a healthy relationship with body image and food among athletes [15,41,51]. Moreover, policy and practice changes should be implemented to improve the sports environment [2,52].

## 5. Conclusions

This work uncovers the relationship between coaches’ leadership styles and athletes’ self-criticism and disordered eating. Transformational leadership styles, such as vision, inspiration, and individualization, play a crucial role in mitigating self-criticism and fostering positive psychological environments in sports. In contrast, negative feedback and passive management styles are associated with increased disordered eating through the mediating role of self-criticism. To address these findings, clubs should improve coaches’ leadership training, by promoting more positive leadership styles. They should also screen athletes for disordered eating and improve access to psychological and nutritional services Lastly, educational, prevention, and intervention programs should be further developed and consistently implemented with athletes, coaches, staff members, and athletes’ support network.

## Figures and Tables

**Table 1:** Correlations between interest variables.

	*M* (*SD*)	1	2	3	4	5	6	7	8	9	10	11	12
1. Disordered Eating	1.16 (1.03)	-											
2. Inadequate Self	2.59 (0.92)	0.57 **	-										
3. Hated Self	0.39 (0.60)	0.48 **	−0.57 **	-									
4. Vision	3.65 (1.03)	−0.06	−0.06	−0.18 *	-								
5. Inspiration	4.00 (0.88)	−0.02	0.06	−0.20 *	0.79 **	-							
6. Instruction	3.90 (0.91)	−0.06	0.07	−0.14	0.74 **	0.85 **	-						
7. Individualization	3.93 (0.89)	−0.08	−0.02	−0.18 *	0.73 **	0.74 **	0.81 **	-					
8. Support	3.12 (0.95)	0.05	0.03	−0.10	0.62 **	0.49 **	0.54 **	0.65 **	-				
9. Positive Feedback	3.61 (0.87)	−0.09	−0.02	−0.13	0.73 **	0.68**	0.69 **	0.70 **	0.55 **	-			
10. Negative Feedback	3.11 (0.95)	0.19 *	0.17 *	0.05	−0.02	0.09	0.05	−0.05	−0.14	−0.02	-		
11. Active Management	3.00 (1.04)	0.04	−0.04	−0.06	0.56 **	0.42 **	0.44 **	0.55 **	0.61 **	0.58 **	−0.18 *	-	
12. Passive Management	2.04 (0.90)	0.21 *	0.09	0.24 *	−0.30 **	−0.44 **	−0.44 **	−0.44 **	−0.22 *	−0.22 *	0.24 *	−0.04	-

* *p* < 0.05; ** *p* < 0.001.

**Table 2:** Mediation analysis of Negative Feedback, Inadequate Self, and Disordered Eating.

Effect	Path	*Β*	*SE*	95% CI		*z*	*p*
				Lower	Upper		
Total	NF > DE	0.21	0.09	0.04	0.38	2.39	0.017
Direct	NF > DE	0.11	0.07	−0.04	0.25	1.43	0.152
Indirect	NF > IS > DE	0.10	0.05	0.00	0.20	2.06	0.039

Note: The table demonstrates that the relationship between Negative Feedback (NF) and Disordered Eating (DE) is fully mediated by self-criticism (Inadequate Self; IS), as indicated by significant indirect and total effects, and an insignificant direct effect.

**Table 3:** Mediation analysis of passive management, Hated Self, and disordered eating.

Effect	Path	*Β*	*SE*	95% CI		*z*	*p*
				Lower	Upper		
Total	PM > DE	0.24	0.09	0.06	0.42	2.58	0.010
Direct	PM > DE	0.11	0.08	−0.06	0.27	1.28	0.200
Indirect	PM > HS > DE	0.13	0.05	0.04	0.22	2.76	0.006

Note. The table demonstrates that the relationship between passive management (PM) and disordered eating (DE) is fully mediated by self-criticism (Hated Self; HS), as indicated by significant indirect and total effects, and an insignificant direct effect.

## Data Availability

The data are not available due to the obtained consent being restrained to this specific study.
